# Utility of local capillary supply indices: Insights from computational image‐based modelling

**DOI:** 10.1113/JP288043

**Published:** 2025-12-05

**Authors:** Abdullah A. Al‐Shammari, Roger W. P. Kissane, Ali Ashkanani, Ashraf Al Madhoun, Fahd Al‐Mulla, Eamonn A. Gaffney, Stuart Egginton

**Affiliations:** ^1^ Department of Mathematics, Faculty of Sciences Kuwait University Shidadiya Kuwait; ^2^ Dasman Diabetes Institute (DDI) Dasman Kuwait; ^3^ Department of Musculoskeletal & Ageing Science University of Liverpool Liverpool UK; ^4^ Department of Community Medicine, Faculty of Medicine Kuwait University Jabriya Kuwait; ^5^ Wolfson Centre for Mathematical Biology, Mathematical Institute University of Oxford Oxford UK; ^6^ School of Biomedical Sciences, Faculty of Biological Sciences University of Leeds Leeds UK

**Keywords:** capillary supply, computational modelling, microvascular geometry, morphometric indices, oxygen delivery, skeletal muscle

## Abstract

**Abstract:**

Muscle oxygenation critically depends on capillary number and distribution within a tissue. Despite the development of numerous morphometric indices to quantify these attributes, many lack sensitivity or validation against local oxygen tension ($P_{O_{2}}$) in muscle fibres, leading to uncertainty about their physiological relevance and utility for gauging adaptive changes. We assessed six local supply indices to determine which best correlated with and predicted calculated fibre $P_{O_{2}}$, using high‐throughput histological analysis and image‐based computational modelling of oxygen transport on digitised rat tibialis anterior sections: capillary contacts (CC*_i_*), individual capillary‐to‐fibre ratio (C:F*_i_*), capillary‐to‐fibre perimeter exchange (CFPE*_i_*), local capillary‐to‐fibre ratio (LCFR*_i_*), local capillary density (LCD*_i_*) and a novel index, D_max,_ *_i_*, which averages the maximum diffusion distances from capillary domains that overlap a fibre. LCD*_i_* and D_max,_ *_i_* exhibited the strongest correlations with fibre $P_{O_{2}}$ (linear correlation: 0.8874 and −0.9054, respectively; *P* < 0.0001). Distance‐correlation analysis confirmed both indices as robust predictors of fibre oxygenation (distance correlation: 0.85 and 0.79, respectively; *P* < 0.0001), demonstrating their ability to capture a curvilinear relationship that plateaus as mean fibre $P_{O_{2}}$ approaches the capillary source $P_{O_{2}}$ (*P* < 0.0001). Partial least squares and tree‐ensemble regressions further identified both indices as the most sensitive and critical predictors of fibre $P_{O_{2}}$ (*P* < 0.001), suggesting they are less likely to miss early pathophysiological or adaptive responses. These findings support the use of theoretically validated morphometric indices in research and clinical applications, to enhance understanding of fundamental muscle physiology and improve the diagnosis and treatment of muscle‐related pathology.

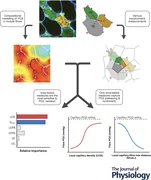

**Key points:**

- Assessing muscle oxygenation is fundamental to understanding muscle function and its adaptive or pathological responses.
- Many indices are used to describe capillary O_2_ supply in skeletal muscle, but their physiological relevance in assessing local oxygenation remains uncertain.
- Using computational modelling of oxygen transport and machine learning, we evaluated six supply indices for their sensitivity in predicting fibre $P_{O_{2}}$.
- Area‐based indices (LCD*_i_* and D_max,_ *_i_*) were identified as the most robust predictors of oxygenation and therefore better descriptors of pathophysiological and adaptive responses.
- These findings are expected to improve both research and clinical assessments of muscle health, streamlining methodologies and enhancing the diagnostic potential for muscle‐related pathologies.

## Graphical abstract

**Abstract figure legend** Local capillary distribution and fibre geometry influence oxygen availability in skeletal muscle. Image‐based modelling of tissue $P_{O_{2}}$ shows that area‐based measures of capillary supply – the local capillary density (LCD*_i_*) and local maximum diffusion distance (D_max,_ *_i_*) – most accurately represent the diffusion constraints imposed by microvascular structure. These indices capture the non‐linear, diffusion‐limited behaviour of fibre oxygenation and outperform traditional integer‐based metrics in predicting local $P_{O_{2}}$. By linking microvascular geometry to oxygen delivery, they provide a physiologically grounded framework for understanding muscle adaptation, performance and pathology.

## Introduction

Muscle oxygenation is essential to support aerobic metabolism and efficient cellular energy production, relying heavily on an intricate network of capillaries for diffusive exchange. Many morphometric indices (i.e. supply indices) have been developed to quantify various aspects of the capillary network, aiming to provide insight into the structural and functional characteristics that affect peripheral oxygen (O_2_) delivery to muscles (Egginton, 1990a). These indices of capillary supply are derived from microscopic images of muscle biopsies, sectioned to assess the distribution and abundance of capillaries in relation to muscle fibres (Al‐Shammari et al., 2019; Egginton & Ross, 1989, 1992; Hepple, 1997). The density and distribution of these capillaries are key determinants of muscle oxygen supply, with diffusive O_2_ delivery influencing muscle performance (especially fatigue resistance), and appropriate responses to altered metabolic demand (Egginton & Hudlická, 2000; Hauton et al., 2015; Hoofd et al., 1985; Kissane, Tickle, et al., 2021; Kissane et al., 2023; Warren et al., 2021).

These supply indices were initially developed to characterise the spatial complexity of a capillary network, which serves as the source of oxygen, in relation to the encased muscle fibres where O_2_ consumption occurs. The rationale is that they should represent the capacity of the microcirculation to supply oxygen, a premise that legitimised their frequent use in studying how muscle responds to various physiological and pathological stimuli (Egginton & Ross, 1992; Loats et al., 1978). Despite their widespread use, validation against direct measurements of local oxygen tension ($P_{O_{2}}$) in fibres remains elusive. While most indices respond to global changes in muscle structure and, to varying degrees, correlate with integrative functional outcomes, only a few exhibit sensitivity to local changes in muscle structure. This is important, as adaptive remodelling of the microcirculation occurs at a length scale of individual fibres (Badr et al., 2003). In contrast to any global response, detecting local changes in oxygen supply capacity requires indices derived from reliable biophysical principles of oxygen diffusion: simply adding more capillaries at random during the process of angiogenesis is inefficient, emphasising the critical role of local feedback signalling (Egginton & Gaffney, 2010). This has not only caused uncertainty among researchers about the relevance and utility of each supply index (Egginton et al., 2020), but has also led to inconsistent results in the literature, for example where glycolytic muscle can have a higher capillary density than that of oxidative muscle (Egginton, 1990a).

A fundamental challenge in the development of any capillary supply index lies in adequately accounting for the complex inter‐related geometry of capillary networks and muscle fibres as first noted by August Krogh, and extensively documented by the use of intravital microscopy (Mendelson et al., 2022). For example, both capillaries and myofibres exhibit irregular and heterogeneous spatial arrangements (Egginton & Ross, 1992; Kissane, Al‐Shammari, et al., 2021), with phenotypic variation in myofibres characterised by asymmetric shapes, non‐uniform cross‐sectional areas and distinct fibre types (Al‐Shammari et al., 2014, 2019). Another challenge is the many indices available, making it unclear which ones adequately describe structural correlates of function (Egginton, 1990a; Egginton et al., 2020). We emphasise here that details of tissue geometry dictate the boundary conditions for the process of oxygen diffusion from capillaries to nearby fibres, thus predominantly impacting local oxygen supply. Determining which index best captures the diffusion process in the intricate network of capillaries and fibres has not been adequately addressed, and may be context‐specific.

We address this issue by evaluating the physiological utility of various morphometric indices through comparison with theoretically computed fibre $P_{O_{2}}$. We integrate high‐throughput histological analysis of a phenotypically heterogeneous muscle (rat tibialis anterior) with image‐based computational modelling to perform a comprehensive assessment of the following local supply indices: capillary contacts, individual capillary‐to‐fibre ratio, capillary‐to‐fibre perimeter exchange, local capillary‐to‐fibre ratio and local capillary density (Al‐Shammari et al., 2019; Egginton, 1990a; Egginton & Ross, 1992; Hepple, 1997; Kissane et al., 2023); and a novel supply index derived from the longest diffusion distance within a capillary supply area or domain, termed local capillary‐to‐fibre maximum distance. These indices are defined in detail in the Methods, and they have been derived for multiple digitised muscle sections for comparison with predictions from the biophysical modelling of oxygen diffusion from multiple capillaries to nearby muscle fibres. This approach allowed us to establish any statistically significant correlation between supply indices and theoretical $P_{O_{2}}$, investigate potential sensitivity in indices to changes in oxygen diffusion and rank indices in terms of their probable importance in predicting theoretical oxygen supply.

This study is the first to attempt such a detailed comparison. Our goal is to identify the most robust indices for quantifying capillary supply and optimising muscle oxygenation, providing a clearer understanding of the utility of these indices. This will aid appropriate choice for investigations into how the microcirculation may underpin adaptive or pathological muscle responses. Resolving the confusion surrounding indices of capillary supply in muscle physiology provides clarity that could lead to more effective interventions and therapies aimed at enhancing muscle function in health and disease.

## Methods

### Ethical approval

Animal sampling followed ethical guidelines set by the United Kingdom Home Office, in line with the 1986 Animal (Scientific Procedures) Act, licence 70/08674, and local animal welfare and ethics committee approval. Four female Sprague‐Dawley rats (Charles River Laboratories, Kingston, PA, USA; weighing 210–240 g) were used in this study. Rats were housed under a 12 h light:dark cycle, and provided with *ad libitum* access to food and water. Following 1 week of acclimation the rats were killed using approved Schedule 1 methods (concussion and cervical dislocation). This work conforms to the ethical requirements outlined by the journal and is presented in accordance with guidelines for animal work (Grundy, 2015; Percie Du Sert et al., 2020).

### Muscle histology and imaging

The tibialis anterior (TA) muscle was excised, trimmed of distal tendons, coated with optimal cutting temperature compound (OCT, Thermo Fisher Scientific, Waltham, MA, USA), snap‐frozen in isopentane cooled by liquid nitrogen, and stored at −80°C. Muscle samples were warmed to −20°C for cryosectioning. Serial sections (10 µm) were cut and fixed on Polysine adhesion slides (VWR International, Radnor, PA, USA), then stored at −20°C until staining. Monoclonal myosin heavy chain (MHC) antibodies were used to simultaneously label two of the three major fibre types (Al‐Shammari et al., 2019), BA‐D5 (1:1000 dilution, Developmental Studies Hybridoma Bank (DSHB)) for type I fibres (slow MHC) with Alexa Fluor 555 Goat Anti‐Mouse IgG (1:1000 dilution) and SC‐71 (1:500 dilution, DSHB) for type IIa (fast oxidative, glycolytic) with Alexa Fluor 488 Rabbit Anti‐Mouse IgG (1:1000 dilution). Laminin (L9393; Sigma, St Louis, MO, USA) was used to identify fibre boundaries, and *Griffonia simplicifolia* lectin I to label capillaries. Five regions of interest (0.145 mm^2^) were imaged across the TA (Fig. 1), two from the oxidative core and three from the glycolytic cortex (Egginton & Ross, 1992). Images were captured with a Qimaging MicroPublisher 5.0 RTV camera on a Nikon Eclipse E600 microscope.

**Figure 1:**
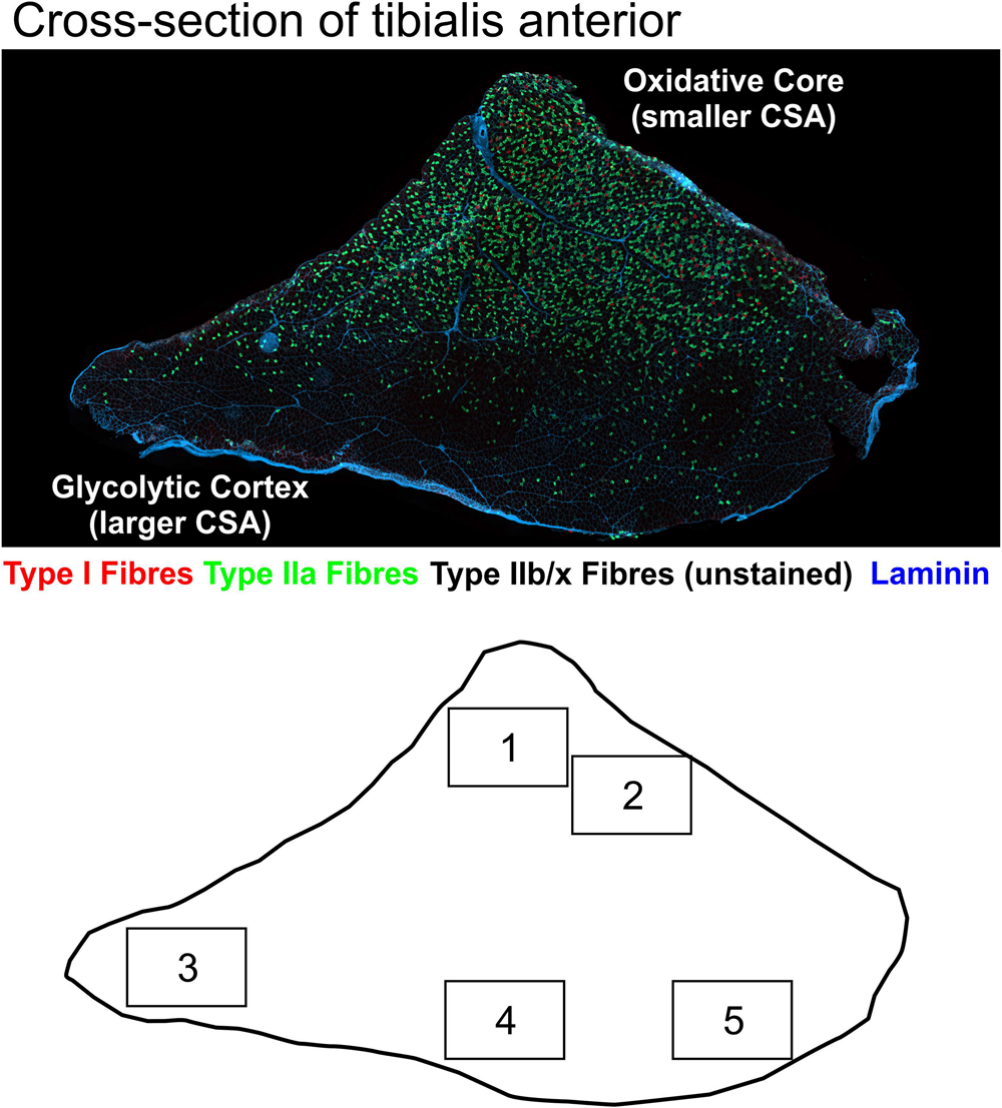
Heterogeneity in muscle phenotype The tibialis anterior was used throughout this study, which is a heterogeneous muscle consisting of a highly oxidative core and glycolytic cortex. Two regions of interest were taken from the core (regions 1 and 2) and three from the cortex of the muscle (regions 3–5); we present data for these two metabolically and functionally distinct compartments to test the utility of indices across a wide range of muscle phenotypes.

### Image segmentation and analysis

A MATLAB‐based graphical user interface, Dtect, was used for semiautomatic segmentation and classification of muscle fibres (Al‐Shammari et al., 2019). Fibre boundaries were identified based on immunostained basal lamina images, with options for manual editing to enhance delineation accuracy. Fibres were classified into types based on colour saturation, with user‐correction capabilities for ambiguous results. Capillary locations were manually marked and linked with adjacent fibres. Data on morphometric statistics were exported for further analysis.

### Morphometric indices

To quantify the structural and functional characteristics of the muscle capillary network, we used the Oxygen Transport Modeller (OTM; Al‐Shammari et al., 2019) to calculate the morphometric indices of interest. With subscript ‘*i*’ indexing the fibre number, these are CC*_i_*, C:F*_i_*, CFPE*_i_*, LCFR*_i_* and LCD*_i_* (refer to Table 1 for formulae and descriptions, and Fig. 2 for graphical illustrations).

**Table 1:** List of morphometric indices of capillary oxygen supply

Capillary supply index	Label	Formula/description	Units
Individual capillary‐to‐fibre ratio	C:F*_i_*	The sum of the fractional contribution of each capillary in contact with fibre *i*	
Capillary contacts	CC*_i_*	Number of capillaries surrounding muscle fibre *i*	
Capillary–fibre perimeter exchange	CFPE*_i_*	$\;\mathrm{CFP}E_{i}=\frac{C:F_{i}}{\mathrm{Perimeter}\;\mathrm{of}\;\mathrm{the}\;ith\;\mathrm{fibre}}$	$\mu m^{-1}$
Fibre region	Fib*_i_*	The region enclosed by the *i*th fibre	
Fibre cross‐sectional area	FCSA*_i_*	The area measure of the *i*th fibre	$\mu m^{2}$
Capillary domain	DOM*_k_*	The region enclosed by the *k*th capillary domain	
Capillary domain area	CDA*_k_*	The area measure of the *k*th capillary domain	$\mu m^{2}$
Local capillary‐to‐fibre ratio of the *i*th fibre	LCFR*_i_*	$\;\mathrm{LCF}R_{i}=\sum_{k=1,\;\text{…},N\mathrm{cap}}\frac{\mathrm{Area}(\mathrm{DO}M_{k}\cap \mathrm{Fi}b_{i})}{\mathrm{CD}A_{k}}$	
Local capillary density of the *i*th fibre	LCD*_i_*	$\;\mathrm{LC}D_{i}=\frac{\mathrm{LCF}R_{i}}{\mathrm{FCS}A_{i}}$	$\mu m^{-2}$
Maximum diffusion distance from a capillary to the *i*th fibre	D*_i_*_,_ *_k_*	The distance from capillary *k* to the farthest point on its capillary domain boundary that falls within fibre *i*	μm
Local capillary‐to‐fibre maximum distance to the *i*th fibre	D_max,_ *_i_*	$\;D_{\max,i}=\frac{1}{N_{\mathrm{overlap},i}}\;\sum_{k}D_{i,k}$	μm

All calculations are based on the selection criteria of capillaries and fibres within the region of interest. ∩ denotes the spatial overlap, *i* refers to the *i*th fibre, ∑*_k_* denotes summing over the list of overlapping capillary domains k=1,2,3,…, and *N*_cap_ is the number of capillaries in the region of interest. $N_{\mathrm{overlap},i}$ is the number of capillaries which have domains that overlap fibre *i*. Further illustrations of the indices are given in Fig. 2, with additional explanation in the Figure legend and the main text.

**Figure 2:**
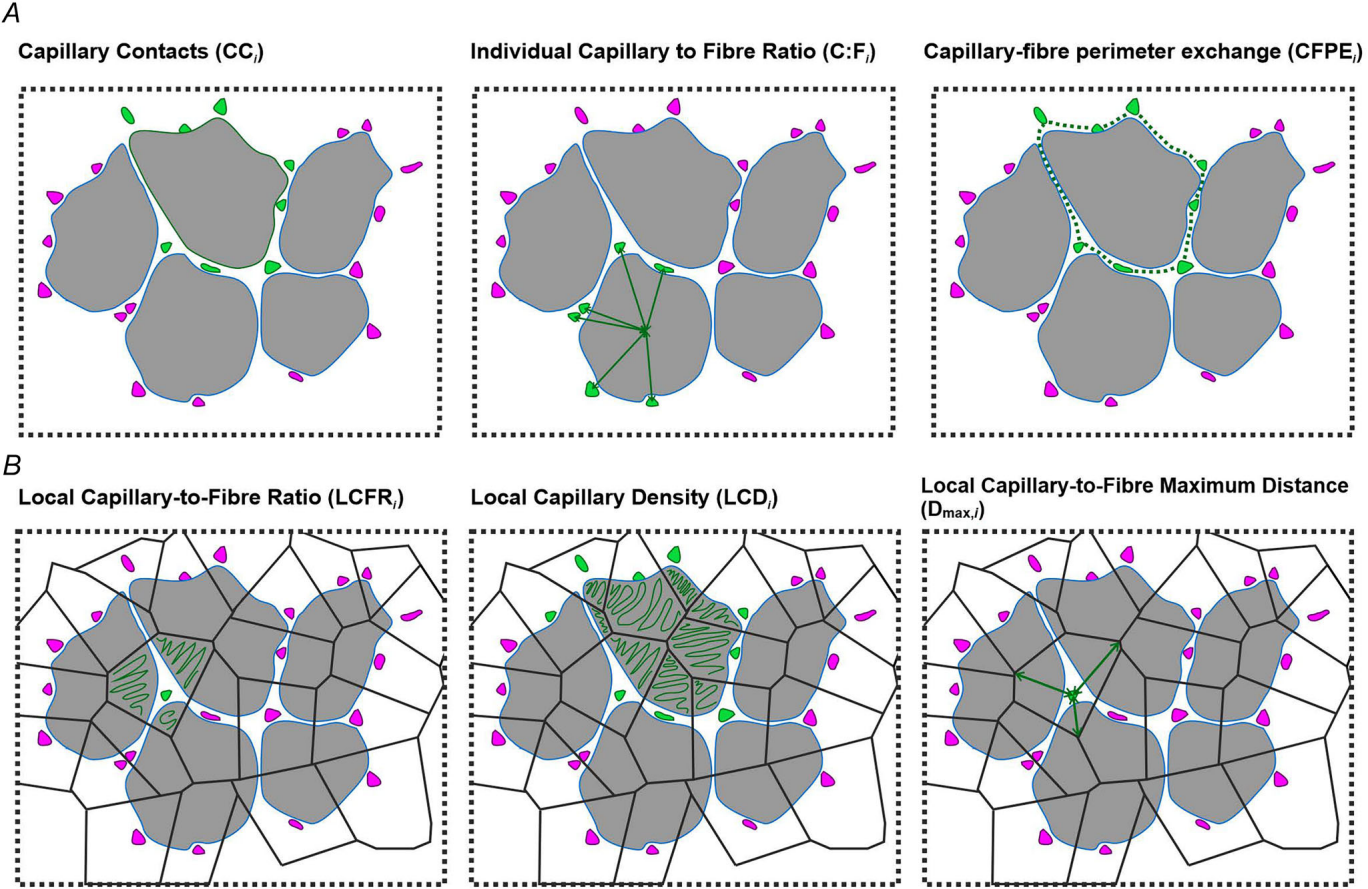
Capillary supply indices *A*, illustrations of integer‐based capillary indices. Left: capillary contacts adjacent to or touching a fibre, as illustrated in green for the upper central fibre. Centre: the individual capillary‐to‐fibre ratio for fibre *i*, and here corresponding to the lower left fibre, is given by the summation of 1/(sharing factor), where the sharing factor is defined in the main text. Right: the fibre perimeter is highlighted, emphasising that the capillary–fibre–perimeter exchange is given for the *i* th fibre by the value of C:F*_i_* divided by the fibre perimeter. *B*, supply area‐derived capillary indices. Left: the capillary domain is depicted by the green shading for the capillary highlighted in green. These are used to compute the local capillary‐to‐fibre ratio. Centre: the local capillary density for the fibre surrounded by green capillaries is the sum of the fractional contributions of the areas of overlapping capillary domains normalised by FCSA. Right: for the green capillary, the associated capillary domain overlaps three nearby fibres. Within each such overlapped fibre, a green line is drawn from the central capillary to the farthest capillary domain vertex. The local capillary‐to‐fibre maximum distance for fibre *i*, denoted by D_max,_ *_i_*, is the average distance of all the green lines that are associated with nearby capillary domains.

Note that for all images and indices, a region of two capillary widths from the image border is excluded from processing, leaving a region of interest where the processing is implemented to remove boundary artefacts from the morphometric indices (Egginton & Ross 1992). The capillary contact (CC*_i_*) is then defined as the number of capillaries in direct contact with a muscle fibre, computed by counting the number of capillaries adjacent to each fibre, illustrated schematically by the green capillaries for the upper central fibre in the left plot of Fig. 2*A*. For this fibre, without loss of generality, indexed as the *i*th fibre, the individual capillary‐to‐fibre ratio (C:F*_i_*) is given by the summation of

$$\frac{1}{\mathrm{Sharing}\;\mathrm{factor}}$$

where ‘sharing factor’ for each capillary denotes the number of fibres each capillary is immediately adjacent to, as also highlighted in the central plot of Fig. 2*A*. The capillary‐to‐fibre perimeter exchange for the *i*th fibre (CFPE*_i_*) is C:F*_i_* divided by the boundary length of the fibre profile and thus measures the capillary supply relative to unit fibre perimeter (see the right plot of Fig. 2*A*). This index was developed to describe the ‘local’ surface area available for exchange between blood and muscle fibres (Hepple, 1997).

Before introducing the next supply index, we define the capillary domain, also known as the capillary supply region (Al‐Shammari et al., 2012, 2014), for the *k*th capillary as the region of tissue points that are closer to this capillary than any other capillary (see the left plot of Fig. 2*B*). This is equivalent to the Voronoi polygon associated with the *k*th capillary in the Voronoi tessellation generated by the point cloud of capillary centres (the spatial set of points/pixels enclosed within this polygon is called a ‘capillary domain’). Throughout, the terms ‘fibre region’ (Fibᵢ) and ‘capillary domain’ (DOM*_k_*) refer to the two‐dimensional (2D) spatial sets used in geometric computations (e.g. overlaps), with their corresponding areas denoted as ‘fibre cross‐sectional area (FCSA)’ and ‘capillary domain area (CDA)’. The local capillary‐to‐fibre ratio of the *i*th fibre (LCFR*_i_*) then denotes the sum of

$$\frac{\mathrm{Area}\;\mathrm{of}\;\mathrm{overlap}\;\mathrm{between}\;kth\mathrm{capillary}\;\mathrm{domain}\;\&\;ith\;\mathrm{fibre}}{kth\;\mathrm{capillary}\;\mathrm{domian}\;\mathrm{area}}\left(*\right)$$

where *k* ranges over all capillaries whose capillary domains overlap the *i*th fibre. This is schematically depicted in the central plot of Fig. 2*B*, where the summation is over the green capillaries and the numerator of (*) is the area of green shading, with the denominator given by the capillary domain area that encloses it (Hoofd et al., 1985). LCFR*_i_* was developed to provide an area‐based measure of local capillary supply (Egginton & Ross, 1992). The local capillary density for the *i*th fibre (LCD*_i_*) is a measure of capillary supply that is normalised by fibre cross‐sectional area (FCSA), obtained by dividing LCFR*_i_* by FCSA of the *i*th fibre (Egginton & Ross, 1992). As a morphometric measure, LCD*_i_* characterises the heterogeneity in both fibre size and fibre type distributions.

Finally, we introduce the *local capillary‐to‐fibre maximum distance* for the *i*th fibre, and denote it by D_max,_ *_i_*. To calculate this local supply index, we first identify all capillaries whose domains overlap with the *i*th fibre. Then, for each overlapping capillary *k*, we calculate the maximum diffusion distance to fibre *i*. We denote this capillary‐to‐fibre distance by D*_i_*_,_ *_k_* and define it as the distance from the centre of capillary *k* to the farthest point on its domain boundary that lies within the *i*th fibre (see the rightmost plot in Fig. 2*B*). Then, to calculate D_max,_ *_i_*, we take the average of all D*_i,k_*, where *k* ranges over all capillaries whose capillary domains overlap the *i*th fibre (i.e. area‐based neighbours)

$$\;D_{\max,i}=\frac{1}{\mathrm{no}.\;\mathrm{of}\;\mathrm{capillaries}\;\mathrm{overlapping}\;\mathrm{the}\;ith\;\mathrm{fibre}}\;\sum_{k}D_{i,k}$$

We note here that a capillary that neighbours multiple fibres may have multiple D*_i,k_* values, each of which is associated with a distinct fibre *i* and therefore should contribute to the calculation of that fibre's D_max,_ *_i_*. For example, in the rightmost plot of Fig. 2*B*, the green capillary has three D*_i,k_* distances (illustrated by the lengths of the three green lines), each corresponding to one of the three fibres that overlap the central capillary domain. By collecting all D*_i,k_* distances for all capillaries, we can calculate D_max,_ *_i_* for each fibre *i* by averaging the lengths of the green lines extended from neighbouring capillaries and ending within that fibre. Hence, this supply index characterises both local diffusion distance and heterogeneity in transversal spacing of nearby capillaries. Collectively, these six indices offer comprehensive insight into the local microvascular architecture and its functional implications for muscle oxygenation.

### Image‐based computational model of tissue $P_{O_{2}}$

The OTM is further used to model and simulate tissue $P_{O_{2}}$ based on digitised muscle sections. Originally developed and described in detail by Al‐Shammari et al. (2019), the OTM was also validated against experimental microvascular morphometry and later shown to reproduce physiologically realistic tissue and fibre $P_{O_{2}}$ distributions. In particular, the model's predictions under high oxygen demand (∼1–5 mmHg; Fig. A4) are consistent with experimental estimates of intracellular $P_{O_{2}}$ during exercise (Poole & Musch, 2023), supporting its physiological validity.

The mathematical model describes the process of transversal oxygen transport in muscle sections via four exchange pathways: O_2_ diffusion across the capillary wall, free O_2_ diffusion according to partial pressure gradients (Fickian diffusion), facilitated diffusion via myoglobin and utilisation within muscle fibres driven primarily by Michaelis–Menten O_2_ consumption (Wilson et al., 1988). We model the muscle tissue as a continuum, 2D space composed of three distinct media or compartments (Al‐Shammari et al., 2014, 2019): capillaries, interstitial space and muscle fibres (type I, IIa, IIb/x). O_2_ exchange between these compartments is assumed to occur at exchange boundaries: the capillary wall and the sarcolemma.

At each capillary–tissue interface, we model O_2_ exchange by a Robin boundary condition where flux of oxygen into the tissue is proportional to the $P_{O_{2}}$ gradient across the capillary wall, thereby accounting for transport resistance at the interface:

$$n_{\mathrm{cap}}\cdot \left[\alpha_{j}D_{j}\nabla p\right]=k\left(p_{\mathrm{cap}}-p\right)$$

where j denotes the tissue compartment where oxygen is transported (fibre types I, IIa or IIb/x, or interstitial space), p denotes tissue $P_{O_{2}}$, α denotes the oxygen solubility, D is the oxygen diffusivity, ‘cap’ denotes the capillary compartment and n is the vector orthogonal (normal) to the boundary, here pointing into the capillary. The proportionality constant, k, is termed the mass transfer coefficient. We note here that this coefficient models a resistance to oxygen transfer across the capillary wall (Federspiel & Popel, 1986). In particular, though not considered in this study, low values of this constant can be used to model sharp $P_{O_{2}}$ drops across the capillary–interstitial barrier of exchange.

At each interstitial‐fibre interface (i.e. sarcolemma), we model O_2_ exchange by assuming continuity in oxygen flux across the sarcolemma (Colburn et al., 2020), which excludes any sarcolemmal resistance to O_2_ transport:

$$n_{\mathrm{int}}\cdot \left[\alpha_{\mathrm{int}}D_{\mathrm{int}}\nabla p\right]=n_{\mathrm{fib}}\cdot \left[\alpha_{\mathrm{fib}}D_{\mathrm{fib}}\nabla p\right]$$

where subscripts int and fib denote the interstitial space and fibre compartments, respectively. In addition, myoglobin contribution to these fluxes is neglected, which is an approximation that does not introduce relevant errors as further detailed in Al‐Shammari et al. (2014), with appeal to the theory of oxygen transport boundary layers (Whiteley et al., 2002).

The primary tissue regions of O_2_ supply and demand are the interstitial space (zero uptake) and muscle fibres (variable uptake), respectively. The interstitial regions are modelled with the assumption that the diffusivity and solubility of O_2_ are equal to those of the neighbouring fibres. Each distinct fibre type is assigned a physiologically informed value for oxygen uptake and myoglobin concentration. Model parameters include oxygen consumption rates, myoglobin concentration, oxygen solubility and diffusivity (refer to Table 2 for model parameters). Overall, the tissue $P_{O_{2}}$ is modelled mathematically in terms of the following non‐linear diffusion equation:

$$\nabla \cdot \;\left[\left(\alpha_{j}D_{j}+\frac{p_{50,\mathrm{Mb}}\cdot C_{j}^{\mathrm{Mb}}\cdot D_{j}^{\mathrm{Mb}}}{{\left[p+p_{50,\mathrm{Mb}}\right]}^{2}}\right)\nabla p\right]=\frac{pM_{0,j}}{p+p_{c}}$$

where $p_{50,\;\mathrm{Mb}}$ is the $P_{O_{2}}$ in tissue at half myoglobin saturation, $C^{\mathrm{Mb}}$ is the concentration of myoglobin, $D^{\mathrm{Mb}}$ is the diffusivity of myoglobin, $p_{c}$ describes the hypoxic threshold of tissue $P_{O_{2}}$ and $M_{0}$ is the fibre‐specific ${\dot{V}}_{O_{2},\max}$.

**Table 2:** Physiological parameters for oxygen transport modelling in homogeneous and mixed muscle

Parameter	Symbol	Uniform phenotype	Fibre type			Units
			I	IIa	IIb/x	
O_2_ demand	$M_{0}$	15.7	15.7	13.82	7.85	10^−5^ ml O_2_/ml s
Mb concentration	$C^{\mathrm{Mb}}$	10.2	10.2	4.98	1.55	10^−3^ ml O_2_/ml
O_2_ solubility	α	3.89 × 10^−5^	3.89 × 10^−5^	3.89 × 10^−5^	3.89 × 10^−5^	ml O_2_/ml mmHg
O_2_ diffusivity	D	2.41 × 10^−5^	2.41 × 10^−5^	2.41 × 10^−5^	2.41 × 10^−5^	cm^2^/s
Mb diffusivity	$D^{\mathrm{Mb}}$	1.73 × 10^−7^	1.73 × 10^−7^	1.73 × 10^−7^	1.73 × 10^−7^	cm^2^/s
Mass transfer coefficient	k	4.0 × 10^−6^	4.0 × 10^−6^	4.0 × 10^−6^	4.0 × 10^−6^	ml O_2_/cm^2 ^mmHg
Intracapillary $P_{O_{2}}$	$p_{\mathrm{cap}}$	30	30	30	30	mmHg
Mb half‐saturated $P_{O_{2}}$	$p_{50,\mathrm{Mb}}$	5.3	5.3	5.3	5.3	mmHg
$P_{O_{2}}$ at half demand	$p_{c}$	0.5	0.5	0.5	0.5	mmHg
Capillary radius		1.8–2.5 × 10^−4^				cm

Default biophysical parameters within Oxygen Transport Modeller, with user versatility to amend parameters (adapted from Al‐Shammari et al., 2014). Note here that we take O_2_ solubility and diffusivity to be uniform for the interstitial spaces and all fibre types, and myoglobin diffusivity to be uniform for all fibre types.

We further assume a no‐flux (boundary condition) at the borders of the digitised muscle section:

$$n_{\mathrm{muscle}}\cdot \;\left[\left(\alpha_{k}D_{k}+\frac{p_{50,\mathrm{Mb}}\cdot C_{k}^{\mathrm{Mb}}\cdot D_{k}^{\mathrm{Mb}}}{{\left[p+p_{50,\mathrm{Mb}}\right]}^{2}}\right)\nabla p\right]=0$$

which introduces a small error that does not impact the results, arising from oxygen transport boundary layers (Whiteley et al., 2002), and further justified in Al‐Shammari et al. (2019).

The full model is then solved computationally in OTM, which utilises the finite elements implementation of the PDE toolbox in MATLAB. Subsequently, the computed $P_{O_{2}}$ is post‐processed to extract summary statistics of individual fibre $P_{O_{2}}$.

### Sensitivity analysis

To test the sensitivity of simulated oxygen distributions to metabolic demand, a parallel set of analyses was conducted, with tissue oxygen consumption ($\dot{M}O_{2}$) increased 10‐fold relative to baseline *M*_0_, approximating an exercise‐like state. The complete image‐based modelling pipeline and subsequent statistical analyses were repeated under these conditions, and the resulting correlations, regressions and variable‐importance metrics are presented as Figs A1, A2, A3.

### Statistical analyses

We aggregated fibres from the region of interest from all muscle sections, consisting of four animals with five regions per animal and multiple fibres per region (*n* = 423 fibres). Unless otherwise stated, statistical significance was set at *P < *0.001. MATLAB version R2023a was used for all data analysis and modelling (The MathWorks Inc., 2023a). All morphometric indices were standardised (centred and scaled to unit standard deviation) prior to data analysis.

Initially, we calculated the linear correlation coefficient (LCC) between each supply index and the simulated mean $P_{O_{2}}$ within the fibres to estimate the strength and direction of the relationship between individual indices and theoretical $P_{O_{2}}$ (see Fig. 3*B*).

**Figure 3:**
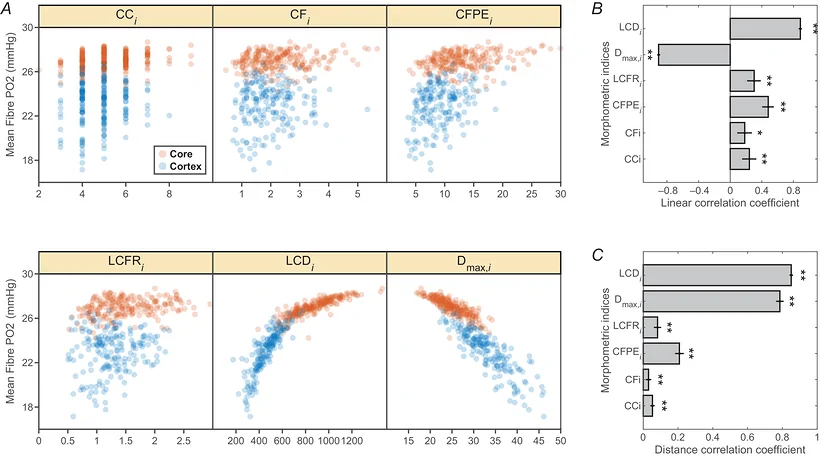
Correlation with fibre $P_{O_{2}}$ under baseline demand *M*_0_ *A*, scatter plots showing the correlation between supply indices (horizontal axes) and theoretical fibre $P_{O_{2}}$ (vertical axis, mmHg). *B*, Pearson linear correlation coefficients. *C*, bias‐corrected distance correlation coefficients. A supplementary analysis with tissue oxygen consumption increased 10‐fold (Fig. A1) yielded proportionally lower fibre $P_{O_{2}}$ values but preserved the qualitative relationships between indices and oxygenation. Horizontal bars indicate 99% confidence intervals. ^*^ *P* < 0.001, ^**^ *P* < 0.0001.

Next, to account for potential co‐linearities between all or several supply indices, we performed a partial least squares regression (PLSR) to detect and estimate the strength of correlation between the six supply indices and the calculated $P_{O_{2}}$. This analysis allows us to estimate the weight and regression coefficient of individual indices in the most explanatory components of the PLSR model (i.e. linear combinations of predictor variables; latent variables), thus providing insights into their individual contributions while accounting for the influence of other indices (see model coefficients in Fig. 4*A*). The PLSR model was resampled using 10‐fold cross‐validation, and the score of variable influence on projection (VIP; Chong & Jun, 2005) was used to measure the importance of each supply index as a predictor of fibre $P_{O_{2}}$. We should emphasise here that such linear analyses are based on the assumption that the underlying relationship between capillary O_2_ supply and local supply indices is linear. However, the local supply must be limited by capillary O_2_ content suggesting that a robust local supply index should show a non‐linear relationship with $P_{O_{2}}$, akin to standard saturation curves. Hence, results from this analysis may only be indicative of general monotonicity (i.e. trend) and should be interpreted with caution.

**Figure 4:**
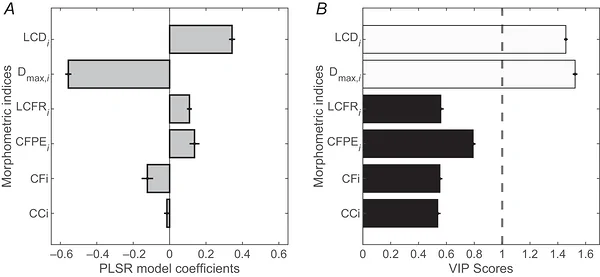
Partial least squares regression under baseline demand *M*_0_ *A*, estimates of the PLSR coefficients indicating the direction and strength of the relationship between supply indices and theoretical fibre $P_{O_{2}}$. *B*, scores of variable influence on projection. A score >1 indicates the predictor variable is important for the PLSR model, whereas a score <1 indicates insignificant influence. Equivalent PLSR results for the high‐demand condition (*M*_0_ × 10) are provided in Fig. A2. Horizontal bars show 99% confidence intervals computed from 100 bootstrapped samples. Cross‐validated *R* ^2^ = 0.88.

To evaluate the non‐linear relationships between supply indices and theoretical $P_{O_{2}}$, we utilised the bias‐corrected distance correlation coefficient (BCDC), which is specifically designed to measure both linear and non‐linear associations (see Fig. 3*C*). This provides a more comprehensive analysis than traditional correlation techniques, which may overlook non‐linear dependencies (Yenigün & Rizzo, 2015). By adjusting for sample size, the BCDC ensures more accurate and less biased correlation estimates. This method involves calculating pairwise distances between data points, forming distance matrices and using these matrices to determine the correlation. Such an approach allows us to detect subtle non‐linear patterns in our data, thus both offering a deeper understanding of the relationship between supply indices and muscle oxygenation and enhancing robustness. Thus, one expects more reliable insight into the physiological relevance of these supply indices.

### Machine learning

We assessed the relative contribution of each supply index to the value and variance of fibre $P_{O_{2}}$ by fitting our data to several machine learning models and conducting a relative importance analysis (Kuhn & Johnson, 2013). This step was crucial to identify which index had the most significant impact on the variation in oxygen transport efficiency within muscle tissue.

We modelled the relationship between supply indices (predictor variables) and theoretical fibre $P_{O_{2}}$ (response variable) by fitting the data to three ensemble models of regression decision‐trees: (1) bagged regression trees (aggregated by bootstrapping), (2) random forests and (3) boosted regression trees (aggregated by least‐squares boosting). A decision tree is a simple model that partitions the dataset (feature space) into regions associated with different predicted values. An ensemble method combines multiple decision trees to improve predictive performance and reduce variance. Not only do these ensemble models offer improved robustness, flexibility and predictive accuracy but they are also well‐suited for capturing complex patterns in the data and handling non‐linear relationships and collinearity. Moreover, their main advantage is the ability to identify important variables by providing various quantitative measures of predictor importance (Kuhn & Johnson, 2013), and hence quantify the sensitivity of each supply index to $P_{O_{2}}$ variance and prediction.

Each regression‐tree ensemble model was fitted to the data using the *fitrensemble* function in the MATLAB Statistics and Machine Learning Toolbox (The MathWorks Inc., 2023b). The option *Bag* was used to train bagged regression trees and random forests. Bagged regression trees employ an ensemble learning approach to enhance prediction accuracy. It uses all features to generate multiple decision trees from bootstrapped samples of the training data and determines the final prediction through averaging individual tree predictions. Random forests extend bagged trees by introducing additional randomness in the selection of predictors at each split during the construction of each decision tree. To train and aggregate boosted regression tress, the option *LSBoost* was used to minimise the mean‐squared error (MSE). This algorithm works by combining multiple weak decision trees into a stronger final model. This is achieved by using a technique called gradient boosting to iteratively improve the model such that, in each step, a new weak tree is added to focus on the errors of the previous ensemble.

The *OptimizeHyperparameters* option in the *fitrensemble* function was specified to automatically identify the optimal hyperparameter configuration for each model. This approach utilises Bayesian optimisation, which is an iterative process that builds a statistical model of the relationship between hyperparameters and model performance. It strategically selects the hyperparameter combination that minimises the cross‐validation MSE. For our machine learning models and dataset, we focused on automatic optimisation of three hyperparameters: the number of ensemble learning cycles (for all models), learning rate (for the boosted trees) and the maximum number of splits (for all models). Here the number of learning cycles is the total number of decision trees making up an ensemble. A larger number of trees can improve the model's accuracy but may also increase computational cost. The learning rate is a parameter that controls how much each tree's prediction contributes to the final ensemble prediction. A smaller learning rate can make the model more robust to overfitting but may require a larger number of trees. The maximum number of splits is the maximal number of branch‐node splits per tree, which controls the depth of each decision tree in an ensemble.

Once hyperparameters were optimised, each model was cross‐validated by holding out 30% of the data for testing and estimates of cross‐validated *R* ^2^ were used for validation. To determine the relative importance of each predictor variable, the *predictorImportance* function was used. This method provides an aggregate measure of variable importance by estimating the variable's contribution to the predictive power of the models. This is achieved by summing changes in the risk due to tree splits on every predictor and dividing the sum by the number of branch nodes. Hence, this measure is directly related to the gain in MSE. A higher importance value indicates that removing the predictor would lead to a larger increase in MSE, suggesting that the predictor is more critical for the model's performance. To account for the influence of potential collinearities between supply indices (e.g. inflated importance, bias in split selection), a recursive feature elimination (Guyon et al., 2002) was implemented *a posteriori* as a check for the robustness of the relative importance of each predictor variable.

We addressed uncertainty in predictor importance values and rankings via a bootstrap resampling procedure. The data were resampled 100 times with replacement, and the machine learning model was refitted to each bootstrap sample. The predictor importance values were calculated for each bootstrap sample, and 99% confidence intervals were constructed based on the distribution of these values. Moreover, predictor rankings were established by looking at the confidence intervals of pairwise differences of these values, with a Bonferroni correction to control the family‐wise error rate.

## Results

### Correlation with fibre $P_{O_{2}}$

Scatter plots (Fig. 3*A*) and correlation coefficients (Fig. 3*B* and *C*) show the correlation between mean theoretical $P_{O_{2}}$ in muscle fibres and local capillary supply indices. We evaluated the correlation using three criteria: (1) strength of trend, (2) level of data dispersion and (3) curvilinearity near capillary‐level $P_{O_{2}}$. Each index showed a statistically significant LCC with fibre $P_{O_{2}}$ (*P* < 0.0001). However, LCD*_i_* and D_max,_ *_i_* exhibited the strongest correlations, 0.8874 and −0.9054, respectively (Fig. 3*B*). Notably, LCD*_i_* displayed the least data dispersion in Fig. 3*A*, suggesting stronger predictive power, higher sensitivity to variation in fibre oxygenation and a more accurate representation of local capillary supply, minimising noise from adjacent fibres. In contrast, the dispersion increases slightly with larger values of D_max,_ *_i_*, suggesting factors other than diffusion distance become more important at such scales.

At high LCD*_i_* and low D_max,_ *_i_* values, mean fibre $P_{O_{2}}$ asymptotically approaches the imposed capillary source $P_{O_{2}}$ (30 mmHg), producing the plateau evident in Fig. 3*A*. This reflects a diffusion‐equilibrium state in which the oxygen gradient between the capillary and fibre becomes minimal, and further increases in local supply cease to elevate intracellular $P_{O_{2}}$.

Figure 3*C* also presents bias‐corrected distance correlations to evaluate the non‐linear relationships (e.g. curvilinearity) between each supply index and theoretical fibre $P_{O_{2}}$. While all indices showed statistically significant distance correlations (*P* < 0.0001), only LCD*_i_* and D_max._ *_i_* exhibited strong correlations, 0.8506 and 0.7865, respectively. This is further confirmed by visual inspection of their scatter plots, as both LCD*_i_* and D_max,_ *_i_* exhibited distinct curvilinear trends that corroborate the earlier observation, confirming that both indices capture the characteristic plateauing behaviour that emerges as mean fibre $P_{O_{2}}$ approaches the capillary source level. The same non‐linear behaviour was quantified by a Hill equation fit of fibre $P_{O_{2}}$ against these indices, showing the characteristic saturation and plateauing pattern (Fig. A5). In contrast, although the other supply indices do show a general positive trend, the plots suggest they are associated with much higher data dispersion but hardly any detectable curvilinearity. This is further confirmed by very low LCC and BCDC, suggesting poor predictive power and sensitivity to local variation in fibre oxygenation. Thus, LCD*_i_* and D_max,_ *_i_* were the most strongly correlated indices with fibre $P_{O_{2}}$.

The TA muscle comprises two metabolically distinct compartments – an oxidative core and a glycolytic cortex – that differ in fibre composition, capillary density and geometry. Despite these marked differences, both regions exhibited the same curvilinear relationships between LCD*_i_*, D_max,_ *_i_* and calculated fibre $P_{O_{2}}$ (Fig. 3, with core and cortex data shown in different colours). This internal consistency demonstrates that the observed relationships are robust across contrasting muscle phenotypes and are probably generalisable to other skeletal muscles and even across species, as similar structural heterogeneity and capillary–fibre geometry are present in both rodent and human skeletal muscle (Egginton, 1990b; Zeller‐Plumhoff et al., 2017).

A high‐demand sensitivity analysis with $\dot{M}O_{2}$ increased 10‐fold (Fig. A1) produced substantially lower absolute fibre $P_{O_{2}}$ values, as expected during exercise, but the overall pattern of relationships among indices and the relative ranking of correlations were preserved. LCD*_i_* and D_max,_ *_i_* continued to show the strongest and most non‐linear associations with calculated fibre $P_{O_{2}}$, confirming the robustness of these relationships across a physiologically relevant range of metabolic rates.

Furthermore, PLSR analysis (see Fig. 4*A* and *B*) indicated that only LCD*_i_* and D_max,_ *_i_* had an important influence on the prediction of fibre $P_{O_{2}}$, with VIP scores >1 and slopes much higher in magnitude compared to the other supply indices (PLSR slope: −0.5644 for D_max,_ *_i_* and 0.3343 for LCD*_i_*). This highlights the robustness and reliability of both LCD*_i_* and D_max,_ *_i_* as predictors of muscle fibre oxygenation.

Equivalent PLSR results obtained under high metabolic demand ($\dot{M}O_{2}$ × 10; Fig. A2) showed consistent patterns, with LCD*_i_* and D_max,_ *_i_* again exhibiting the largest variable‐influence scores (VIP > 1) and regression coefficients in both magnitude and direction. Thus, the predictive dominance of these indices persisted regardless of metabolic load.

### Relative predictive importance of supply indices

The predictive power and sensitivity to $P_{O_{2}}$ variation were further assessed and ranked in Fig. 5, which presents the results of tree‐ensemble regression analysis as values of relative importance of supply indices as predictors of theoretical fibre $P_{O_{2}}$. Notably, both LCD*_i_* and D_max,_ *_i_* exhibited significantly higher relative predictive importance of fibre oxygenation in comparison to the other indices (*P* < 0.001), which showed only marginal influence. LCD*_i_* emerged as the most critical predictor in the TA muscle and both its core and cortex regions, whereas D_max,_ *_i_* ranked second. In contrast, although CFPE*_i_* ranked third in $P_{O_{2}}$ predictive importance, it exhibited a much lower value of relative predictive importance. While the relative ranking of indices was consistent across compartments, the absolute magnitude of importance differed between them, because each dataset was trained and normalised independently for each compartment. The higher relative‐importance values of LCD*_i_* and D_max,_ *_i_* observed in the cortical region (Fig. 5) therefore reflect differences in the internal normalisation of the independently trained machine‐learning models rather than physiological variation. Therefore, relative‐importance values are not directly comparable between panels. The resemblance between whole‐muscle and cortical results primarily reflects a relative volume effect of the cortical compartment within the TA.

**Figure 5:**
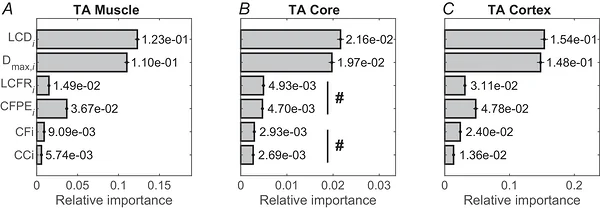
Relative importance of capillary supply indices under baseline demand *M*_0_ Capillary supply indices ranked, in the bagged regression‐trees model, according to their predictive strength of and sensitivity to changes in theoretical fibre oxygenation. Similar rankings (not shown here) were also observed for the other two ensemble models (random forests and boosted regression trees). To account for potential collinearities, all rankings were confirmed by recursive feature elimination. Data are presented for (*A*) the whole tibialis anterior muscle (cross‐validated *R* ^2^ = 0.961), and for the two individual compartments, (*B*) the oxidative core (cross‐validated *R* ^2^ = 0.864) and (*C*) glycolytic cortex (cross‐validated *R* ^2^ = 0.916). Corresponding results for the high‐demand conditions (*M*_0_ × 10) are shown in Fig. A3. Horizontal error bars show 99% confidence intervals computed from 100 bootstrapped samples. Multiple comparisons were performed *post hoc*, with Bonferroni correction (*n* = 15). **^#^** Pairs that are not statistically different in terms of relative importance (*P* > 0.001). All other pairs of indices are significantly different in terms of relative importance (*P* < 0.001). Each dataset had a machine‐learning model that was trained and normalised independently; therefore, relative‐importance values are not directly comparable between panels.

Tree‐ensemble regression analyses performed under high metabolic demand ($\dot{M}O_{2}$ × 10; Fig. A3) yielded essentially the same hierarchical ranking of supply indices as observed in resting simulations. LCDᵢ remained the most critical predictor, followed by D_max,_ *_i_*, while the remaining indices showed only minor contributions to the variance in fibre $P_{O_{2}}$.

## Discussion

In this study, we investigated the relationship between commonly used morphometric indices of local capillary supply and muscle fibre $P_{O_{2}}$. While many morphometric indices have been developed to assess muscle capillarisation, their ability to reflect true physiological oxygenation is unclear due to the lack of validation against direct $P_{O_{2}}$ measurements. In particular, some indices lack sufficient sensitivity to physiological changes (Egginton, 1990a; Van Vossel et al., 2024). To address this gap, we used high‐throughput muscle histological analysis and image‐based computational modelling to compute theoretical fibre $P_{O_{2}}$ in rat TA muscle sections, thereby allowing direct statistical assessment of indices of local capillary supply. By comparing these values to traditional and more recently developed supply indices, we aimed to identify the most robust approach for quantifying capillary supply and improve the structural basis for optimising muscle oxygenation.

Our findings demonstrate that area‐based indices, LCD*_i_* and D_max_,*_i_*, have the strongest correlation with calculated fibre $P_{O_{2}}$, and are the most sensitive and predictive measures of local transport of oxygen. The other, integer‐based supply indices showed weaker correlation, sensitivity and predictiveness. This is probably because commonly used indices, such as CFPE*_i_*, may not fully capture the detailed geometry of the microvascular architecture that limits local oxygen transport (Egginton, 1990a). For example, it could miss subtle or functionally relevant variations in capillary distribution, such as changes to capillary spacing (Degens et al., 2006; Egginton & Gaffney, 2010; Hendrickse & Degens, 2019; Zeller‐Plumhoff et al., 2017), or functional recruitment that can occur with resistance training (Van Vossel et al., 2024). In contrast, the strong correlation between LCD*_i_* and D_max,_ *_i_* with muscle fibre $P_{O_{2}}$ suggests these area‐based indices may be better suited to predict muscle oxygenation and potentially even muscle performance during aerobic exercise. Importantly, these relationships were consistent across the two distinct compartments of the TA (core and cortex), indicating that the underlying geometric principles are robust across contrasting muscle phenotypes and probably extend to other mammalian muscles. This robustness also holds when accounting for fibre‐type myoglobin differences. Indeed, variations in myoglobin concentration primarily influence the absolute magnitude of fibre $P_{O_{2}}$, with facilitation becoming appreciable only at very low intracellular $P_{O_{2}}$ (<5 mmHg). However, such differences do not materially alter the relationships between $P_{O_{2}}$ and the capillary‐supply indices (LCD*_i_* and D_max,_ *_i_*), which remain qualitatively consistent across fibre types and metabolic conditions.

To further test the physiological plausibility of our findings under exercise‐like conditions, we repeated all simulations with tissue oxygen consumption increased 10‐fold. Although this resulted in markedly lower absolute fibre $P_{O_{2}}$ values – consistent with experimental estimates during muscle activity (Poole & Musch, 2023) – the relationships and rankings among supply indices remained unchanged with LCDᵢ and D_max_,ᵢ retaining their predictive dominance (Figs A1, A2, A3). This confirms that our central findings are independent of metabolic state and reflect intrinsic geometric determinants of local oxygen transport.

Not surprisingly, our results revealed that only LCD*_i_* and D_max_,*_i_* exhibit a curvilinear relationship with theoretical fibre $P_{O_{2}}$. This non‐linear behaviour is further illustrated by a Hill equation fit of calculated fibre $P_{O_{2}}$ against these supply indices (Fig. A5), which clearly demonstrates the saturation and plateauing effects observed in the scatter plots. Because local oxygen supply is inherently limited by the amount of oxygen available within capillaries, an adequate supply index must exhibit this curvilinearity as it reflects a physiological constraint. This feature is crucial for accurately modelling oxygen diffusion and understanding muscle oxygenation dynamics.

These findings highlight the limitations of many capillary supply indices in common usage, and the inherent confusion that may arise from comparing studies where conclusions are based on different indices. For example, the finding that a 10 week knee extensor resistance training did not correspond with significant changes in capillarisation (Van Vossel et al., 2024) suggests the use of a broader set of local supply indices (including LCD*_i_* and D_max,_ *_i_*) could provide a more complete picture and help clarify inconsistencies across studies that use different evaluation metrics. Additionally, the discrepancies observed in previous studies, particularly those highlighting that global capillary density alone is not a sufficient parameter to describe the functional capacity of the capillary bed (Egginton, 1990a; Hendrickse & Degens, 2019; Zeller‐Plumhoff et al., 2017), underscore the necessity for using indices that are both biophysically sound and empirically validated. Our study suggests that area‐based indices can offer more accurate assessments of muscle oxygenation and inform the implications of various interventions or pathologies on muscle structure and function. For example, a tight link was found between local capillary supply and fatigue resistance (Tickle et al., 2020), while changes in LCD were tightly linked to functional decline and recovery following spinal cord injury (Warren et al., 2021). Additionally, we have previously found changes in capillary domain areas are associated with functional improvements in muscle performance (fatigue resistance) after interventions to promote angiogenesis (Kissane, Tickle, et al., 2021). Correlation between morphometric measures and muscle tissue oxygenation has further confirmed that the major measure to consider is the distribution of capillaries, or red blood cells (Zeller‐Plumhoff et al., 2017).

In conclusion, this study emphasises the importance of validating morphometric indices against theoretical models of oxygen transport. The strong predictive power of LCD*_i_* and D_max,_ *_i_* offers a clearer understanding of muscle microvascular architecture and its functional implications. Future research may refine these indices and explore their applications across different muscle types and pathological conditions to enhance our understanding of muscle physiology and improve clinical outcomes.

### Potential limitations

This study employed a 2D (planar) analysis to assess muscle oxygen transport. While 3D imaging techniques are gaining traction, offering a higher dimensional representation of capillary networks (Zeller‐Plumhoff et al., 2017), our model effectively captures the key physiological processes influencing local oxygen diffusion. Extending these analyses into three dimensions represents a natural next step for future work, particularly in resolving axial capillary tortuosity and the connectivity between transverse capillary domains.

Recent work has challenged the traditional assumption of appreciable intracellular oxygen gradients and diffusion‐limited metabolism, proposing instead that mitochondrial organisation and intracellular oxygen transport may function as an integrated ‘powergrid’ that maintains relatively uniform $P_{O_{2}}$ across the fibre (Poole & Musch, 2023). While such frameworks may describe diffusion‐sufficient conditions, our modelling explicitly targets scenarios where oxygen diffusion becomes limiting – for example, in tissues with reduced capillary density, impaired perfusion or elevated metabolic demand. Under these circumstances, the assumption of spatially uniform intracellular $P_{O_{2}}$ may not hold, and diffusion gradients become functionally significant. Incorporating explicit diffusion therefore enables quantification of how microvascular geometry and density influence both intracellular and interstitial oxygen availability. Consistent with experimental observations (Poole & Musch, 2023), our high O_2_‐demand simulations predict intracellular $P_{O_{2}}$ values that fall within the physiological range measured during muscle contraction and elevated metabolic demand (Fig. A4). Future developments could integrate finer‐scale mitochondrial representations to explore how such ‘powergrid’ mechanisms interact with oxygen diffusion and capillary geometry to sustain energetic homeostasis (Al‐Shammari, 2014; Poole & Musch, 2023).

Additionally, the adoption of a Fickian‐facilitated diffusion model may not fully account for the alternative fibre‐interface–diffusion‐limitation theory (Hepple, 1997). However, experimental evidence suggests the primary diffusion resistance occurs at the capillary wall (Gayeski & Honig, 1988; Poole et al., 2022), a factor that is already integrated into our model through the capillary permeability parameter, κ. This limitation of transport may become more relevant at extremely low $P_{O_{2}}$ values (∼0.5 mmHg), requiring further investigation. The contrasting hypothesis suggesting high resistance to O_2_ diffusion at the sarcolemmal interface, and the related inclusion of fibre perimeter in the supply index CFPE*_i_* (Hepple, 1997), has yet to be satisfactorily explored. Moreover, the parameterised modelling of three levels of resistance to local tissue oxygen transport – capillary wall, spatial diffusion and fibre membrane – presents substantial experimental challenges, particularly in quantifying membrane permeability in a manner analogous to estimating the capillary mass transfer coefficient (Federspiel & Popel, 1986). Future studies may explore these complexities using more advanced techniques.

## Additional information

## Competing interests

The authors declare that they have no competing interests.

## Author contributions

A.A.Al‐S., R.K. and S.E. conceived and designed the research. A.A.Al‐S. drafted the manuscript. R.K. performed animal tissue preparation, histological staining and imaging, and prepared digitised images of muscle sections. A.A.Al‐S. and A.A. conducted high‐throughput computational modelling. A.A.Al‐S. performed data analysis. A.A.Al‐S., R.K., E.A.G. and S.E. interpreted the results. A.A.Al‐S., R.K., E.A.G., A.M., F.M. and S.E. helped to draft and revise the manuscript. All authors gave approval for final publication.

## Funding

The authors are grateful to the School of Biomedical Sciences, University of Leeds, for provision of a scholarship to R.W.P.K. This research was supported by a British Heart Foundation Project Grant (PG/14/15/30691).

## Supporting information




Peer Review History

Dataset containing all morphometric indices and corresponding oxygen partial pressure values for all individual muscle fibres analysed in this study.

## Figures and Tables

**Figure A1:**
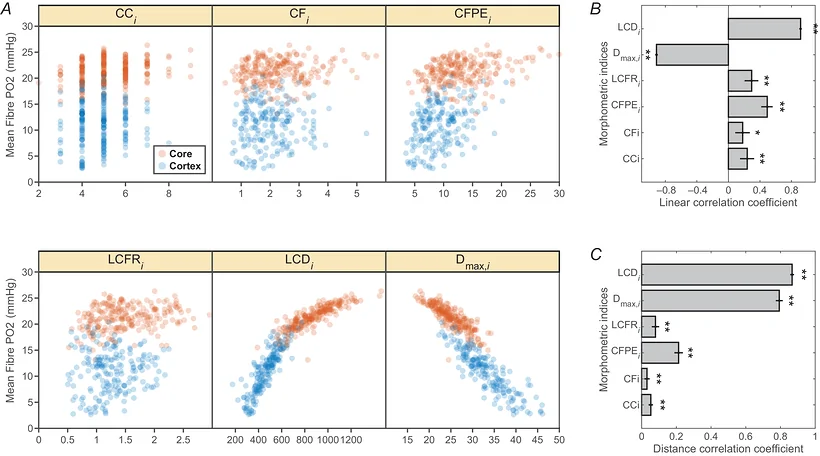
Correlation with fibre $P_{O_{2}}$ under high demand Scatterplots and correlation analyses equivalent to Fig. 3, generated from image‐based simulations with tissue $\dot{M}O_{2}$ increased 10‐fold to model fibre $P_{O_{2}}$ distribution under exercise conditions. The higher metabolic rate substantially reduced the absolute fibre $P_{O_{2}}$ values but preserved the qualitative behaviour of all relationships between supply indices and oxygenation. LCDᵢ and D_max,_ *_i_* remained the strongest and most predictive of fibre $P_{O_{2}}$, capturing non‐linear behaviours, and thus confirming that their explanatory power is maintained across a physiologically relevant range of oxygen demands.

**Figure A2:**
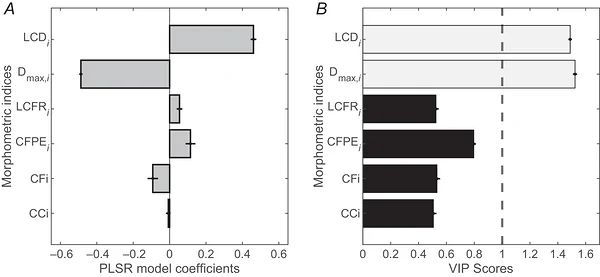
Partial least squares regression under high demand Estimates of the PLSR coefficients – equivalent to Fig. 4 – under a 10‐fold increase in tissue $\dot{M}O_{2}$. LCDᵢ and D_max,_ *_i_* continue to exhibit the largest variable‐influence scores and regression coefficients, confirming their dominant predictive role.

**Figure A3:**
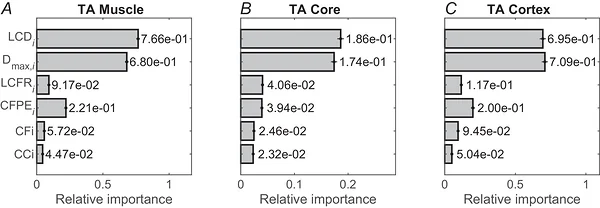
Relative importance of capillary supply indices under high demand Estimates of relative importance of supply indices in predicting calculated fibre $P_{O_{2}}$ – equivalent to Fig. 5 – under a 10‐fold increase in tissue $\dot{M}O_{2}$. The ranking of indices and their relative contributions to fibre $P_{O_{2}}$ variance are essentially unchanged compared with baseline metabolic rate *M*_0_.

**Figure A4:**
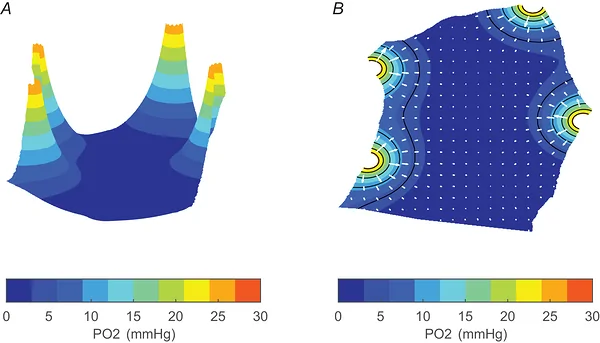
Intracellular $P_{O_{2}}$ distributions under high metabolic demand *A*, spatial distribution of simulated intracellular $P_{O_{2}}$ at 10‐fold tissue $\dot{M}O_{2}$ shows markedly lower mean values (≈1–5 mmHg), consistent with experimental estimates for exercising muscle (Poole & Musch, 2023). *B*, the presence of mild intracellular gradients (white arrows) reflects diffusion limitation at elevated metabolic demand, demonstrating that the model accurately captures both low‐$P_{O_{2}}$ regimes and the geometric determinants of oxygen transport.

**Figure A5:**
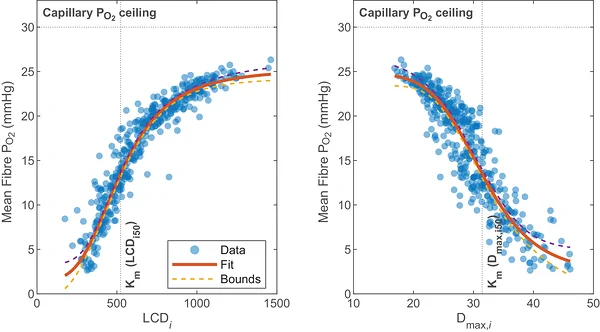
Non‐linear plateauing of mean fibre $P_{O_{2}}$ Scatter plots of calculated fibre $P_{O_{2}}$ against (*A*) LCD*_i_* and (*B*) D_max,_ *_i_* fitted with a general Hill equation to quantify the observed non‐linearity and plateauing behaviour. Each fit includes 99% confidence bounds for the estimated parameters (LCD*_i_* fit: *R* ^2^ = 0.917; D_max,_ *_i_* fit: *R* ^2^ = 0.846). Both curves show a steep increase in fibre $P_{O_{2}}$ with increasing local capillary supply, followed by a pronounced levelling‐off as $P_{O_{2}}$ approaches the capillary source value (∼30 mmHg). This saturation reflects diffusion equilibrium at the capillary–tissue interface and quantitatively supports the curvilinear trends shown in Fig. 3*A*.

## Data Availability

All morphometric and simulation data generated for individual muscle fibres have been compiled into a single spreadsheet and are provided as Supporting information.
