# Supplementary material for: Utility of local capillary supply indices: Insights from computational image‐based modelling
Source: J Physiol. 2025 Dec 5;604(19):8219–36. doi: 10.1113/JP288043 (PMC13628553; doi:10.1113/JP288043)
Supplement: Supplementary file 1 [file TJP-604-8219-s002.pdf]

The Journal of Physiology

Utility of local capillary supply indices: insights from computational image-based 1 modelling

Abdullah A Al-Shammari, Roger W.P Kissane, Ali Ashkanani, Ashraf Madhoun, Fahd Al-Mulla, Eamonn A Gaffney, and Stuart Egginton
DOI: 10.1113/JP288043

Corresponding author(s): Abdullah Al-Shammari (abdullah.alshammari@ku.edu.kw)

The referees have opted to remain anonymous.

Review Timeline:

Submission Date:	06-Nov-2024
Editorial Decision:	18-Dec-2024
Revision Received:	17-Oct-2025
Accepted:	17-Nov-2025

Senior Editor: Natalia Trayanova

Reviewing Editor: T Alexander Quinn

Transaction Report:

(Note: With the exception of the correction of typographical or spelling errors that could be a source of ambiguity, letters and reports are not edited. Depending on transfer agreements, referee reports obtained elsewhere may or may not be included in this compilation. Referee reports are anonymous unless the Referee chooses to sign their reports.)

Dear Dr Egginton,

Re: JP-RP-2024-288043 "Utility of local capillary supply indices: insights from computational image-based modelling" by Abdullah A Al-Shammari, Roger W.P Kissane, Ali Ashkanani, Ashraf Madhoun, Fahd Al-Mulla, Eamonn A Gaffney, and Stuart Egginton

Thank you for submitting your manuscript to The Journal of Physiology. It has been assessed by a Reviewing Editor and by 2 expert referees and we are pleased to tell you that it is potentially acceptable for publication following satisfactory major revision.

Please advise your co-authors of this decision as soon as possible.

The referee reports are copied at the end of this email.

Please address all the points raised and incorporate all requested revisions or explain in your Response to Referees why a change has not been made. We hope you will find the comments helpful and that you will be able to return your revised manuscript within 9 months. If you require longer than this, please contact journal staff: jp@physoc.org. Please note that this letter does not constitute a guarantee for acceptance of your revised manuscript.

Your revised manuscript should be submitted online using the link in your Author Tasks: Link Not Available. This link is accessible via your account as Corresponding Author; it is not available to your co-authors. If this presents a problem, please contact journal staff (jp@physoc.org). Image files from the previous version are retained on the system. Please ensure you replace or remove any files that are being revised.

If you do not wish to submit a revised version of your manuscript, you must inform our journal staff (jp@physoc.org) or reply to this email to request withdrawal. Please note that a manuscript must be formally withdrawn from the peer review process at one journal before it may be submitted to another journal.

TRANSPARENT PEER REVIEW POLICY: To improve the transparency of its peer review process, The Journal of Physiology publishes online as supporting information the peer review history of all articles accepted for publication. Readers will have access to decision letters, including Editors' comments and referee reports, for each version of the manuscript, as well as any author responses to peer review comments. Referees can decide whether or not they wish to be named on the peer review history document.

ABSTRACT FIGURES: Authors are expected to use The Journal's premium BioRender account to create/redraw their Abstract Figures. Information on how to access this account is here: https://physoc.onlinelibrary.wiley.com/journal/14697793/biorender-access.

This will enable Authors to create and download high-resolution figures. If authors have used the free BioRender service, they can use the instructions provided in the link above to download a high-resolution version suitable for publication.

The link provided should only be used for the purposes of this submission. Authors will be charged for figures created on this account if they are not related to this manuscript submission.

LANGUAGE EDITING AND SUPPORT FOR PUBLICATION: If you would like help with English language editing, or other article preparation support, Wiley Editing Services offers expert help, including English Language Editing, as well as translation, manuscript formatting, and figure formatting at www.wileyauthors.com/eeo/preparation. You can also find resources for Preparing Your Article for general guidance about writing and preparing your manuscript at www.wileyauthors.com/eeo/prepresources.

RIGOUR AND REPRODUCIBILITY: We recommend authors consult The Journal's Rigour and Reproducibility requirements to ensure that their manuscript is suitable for publication: https://physoc.onlinelibrary.wiley.com/pb-assets/hub-assets/physoc/documents/TJP-Rigour-and-Reproducibility-Requirements.pdf

REVISION CHECKLIST:

Check that your Methods section conforms to journal policy: https://jp.msubmit.net/cgi-bin/main.plex?form_type=display_requirements#methods.

Check that data presented conforms to the statistics policy: https://jp.msubmit.net/cgi-bin/main.plex?form_type=display_requirements#statistics.

Upload a full Response to Referees file. To create your 'Response to Referees': copy all the reports, including any comments from the Senior and Reviewing Editors, into a Microsoft Word, or similar, file and respond to each point, using font or background colour to distinguish comments and responses and upload as the required file type.

Please upload two versions of your manuscript text: one with all relevant changes highlighted and one clean version with no changes tracked. The manuscript file should include all tables and figure legends, but each figure/graph should be uploaded as separate, high-resolution files.

You may also upload:

- 'Potential Cover Art' for consideration as the issue's cover image
- Appropriate Supporting Information (Video, audio or data set: see https://jp.msubmit.net/cgi-bin/main.plex?form_type=display_requirements#supp).

We look forward to receiving your revised submission.

If you have any queries, please reply to this email and we will be pleased to advise.

Yours sincerely,

Natalia Trayanova
Senior Editor
The Journal of Physiology

REQUIRED ITEMS

- Include a Key Points list in the article itself, before the Abstract.

- Author photo and profile. First or joint first authors are asked to provide a short biography (no more than 100 words for one author or 150 words in total for joint first authors) and a portrait photograph. These should be uploaded and clearly labelled together in a Word document with the revised version of the manuscript. See Information for Authors for further details.

- Your manuscript must include a complete Additional Information section, including competing interests; funding; author contributions and acknowledgements.

- The Journal of Physiology funds authors of provisionally accepted papers to use the premium BioRender site to create high resolution schematic figures. Follow this link and enter your details and the manuscript number to create and download figures. Upload these as the figure files for your revised submission. If you choose not to take up this offer, we require figures to be of similar quality and resolution. If you are opting out of this service to authors, state this in the Comments section on the Detailed Information page of the submission form. The link provided should only be used for the purposes of this submission. Authors will be charged for figures created on this premium BioRender account if they are not related to this manuscript submission.

- Please upload separate high-quality figure files via the submission form.

- Please ensure that the Article File you upload is a Word file.

- Papers must comply with the Statistics Policy: https://jp.msubmit.net/cgi-bin/main.plex?form_type=display_requirements#statistics.

In summary:

- If n {less than or equal to} 30, all data points must be plotted in the figure in a way that reveals their range and distribution. A bar graph with data points overlaid, a box and whisker plot or a violin plot (preferably with data points included) are acceptable formats.

- If n > 30, then the entire raw dataset must be made available either as supporting information, or hosted on a not-for-profit repository, e.g. FigShare, with access details provided in the manuscript.

- 'n' clearly defined (e.g. x cells from y slices in z animals) in the Methods. Authors should be mindful of pseudoreplication.

- All relevant 'n' values must be clearly stated in the main text, figures and tables.

- The most appropriate summary statistic (e.g. mean or median and standard deviation) must be used. Standard Error of the Mean (SEM) alone is not permitted.

- Exact p values must be stated. Authors must not use 'greater than' or 'less than'. Exact p values must be stated to three significant figures even when 'no statistical significance' is claimed.

- A Data Availability Statement is required for all papers reporting original data. This must be in the Additional Information section of the manuscript itself. It must have the paragraph heading 'Data Availability Statement'. All data supporting the results in the paper must be either: in the paper itself; uploaded as Supporting Information for Online Publication; or archived in an appropriate public repository. The statement needs to describe the availability or the absence of shared data. Authors must include in their statement: a link to the repository they have used, or a statement that it is available as Supporting Information; reference the data in the appropriate sections(s) of their manuscript; and cite the data they have shared in the References section. Whenever possible, the scripts and other artefacts used to generate the analyses presented in the paper should also be publicly archived. If sharing data compromises ethical standards or legal requirements then authors are not expected to share it, but must note this in their statement. For more information, see our Statistics Policy.

- Please include an Abstract Figure file, as well as the Figure Legend text within the main article file. The Abstract Figure is a piece of artwork designed to give readers an immediate understanding of the research and should summarise the main conclusions. If possible, the image should be easily 'readable' from left to right or top to bottom. It should show the physiological relevance of the manuscript so readers can assess the importance and content of its findings. Abstract Figures should not merely recapitulate other figures in the manuscript. Please try to keep the diagram as simple as possible and without superfluous information that may distract from the main conclusion(s). Abstract Figures must be provided by authors no later than the revised manuscript stage and should be uploaded as a separate file during online submission labelled as File Type 'Abstract Figure'. Please also ensure that you include the figure legend in the main article file. All Abstract Figures should be created using BioRender. Authors should use The Journal's premium BioRender account to export high-resolution images. Details on how to use and access the premium account are included as part of this email.

- Please include a full title page as part of your main article (Word) file, which should contain the following: title, authors, affiliations, corresponding author name and contact details, keywords, and running title.

Reviewing Editor's comments:

Your paper has been reviewed by two experts in the field. They felt the work is important and has potential for influence within the field. They did, however, have suggestions for improving the overall quality of the work. Please submit a revision that addresses these comments, along with a point-by-point response to the reviewers.

Referee #1:

This manuscript seeks to assess what capillary indices give the best reflection of tissue pO2. They used model calculations to assess the tissue pO2. The manuscript comes to the conclusion that the local capillary density and a new parameter, the maximal diffusion distance from a capillary in a capillary domain overlapping a fibre, are the best indicators of tissue oxygenation.

Fibre pO2 seems to be high in, for instance, figure 3, for a working muscle, where it has been suggested that the pO2 within a fibre maybe close to 1.5 mmHg during exercise, even when capillary pO2 is 30 mmHg Also, suggestions have been made that fibre pO2 may not be best modelled with the Krogh cylinder, but rather the Hill model and that intracellular oxygen profiles are rather flat and metabolism not oxygen diffusion limited, as for instance realised by a 'mitochondrial powergrid' (e.g. review by David C Poole, Timothy I Musch in: Function, 4(3):zqad013, 2023: Capillary-Mitochondrial Oxygen Transport in Muscle: Paradigm Shifts). While I do not necessarily agree with the concepts proposed in that review, it would be good to discuss this and give some thoughts would affect your pO2 calculations and impacts of capillary indices on pO2.

The manuscript it well-written and a clear rationale for the work is given.

Abstract

Line 5: There was not so much a comparison of capillary indices with fibre pO2, but rather which parameter(s) correlated best with the calculated fibre pO2.

Line 6 from bottom: Do you mean 'venous' capillary-level pO2?

Methods

Clearly described method. However, I do suggest changing the name 'fibre region' into the more commonly used 'fibre cross-sectional area'. I understand that 'area' is mentioned lower in the tables, but perhaps it is making matters unnecessarily complicated, and it could also be referred to as FCSA. Same for 'capillary domain' where I think you could as well write 'capillary domain area'.

Can you discuss briefly how Mb in type IIa cells would affect the calculations? In table 2 it is suggested that there is no Mb in type IIa cells.

Referee #2:

This manuscript describes the results of a study that utilizes new histological data on rat skeletal muscle (tibialis anterior, TA) and computational modeling of oxygen (O2) transport (previously described OTM model) to evaluate various capillary-based measures of O2-transport efficiency. Both standard statistical and machine learning (ML) approaches are used to compare estimated muscle fiber oxygenation (O2 partial pressure, PO2) with predictions of the efficiency measures, which are based on 2D muscle sections where capillary, muscle fiber, and interstitial compartments are considered. Six measures are evaluated and two (the second of which I believe is novel) are found to strongly correlate with PO2, in particular, local capillary density (LCDi, positively correlated) and local capillary-fiber maximum distance (Dmaxi, negatively correlated). LCDi and D_maxi are shown to be informative both by standard statistics and several machine learning models, so the results are quite strong. I believe these results could have a significant impact in the field, and only have a few comments for the authors to consider, detailed below.

Major Comments:

1. According to Fig.3, the results presented focus on muscle fibers that have PO2 levels of 18mmHg or above. Given the assumed Pcrit of 0.5mmHg, this means that O2 consumption is barely impacted by decreased muscle PO2 (97% or greater of max). This presumably corresponds to healthy resting muscle, but it's not clear how generally applicable the results are to, for example, light or moderate exercise. Have the authors considered either increasing O2 consumption or decreasing the input capillary PO2 of 30mmHg in the computational model?

2. The description of the OTM model on P.9 (second paragraph) could be a bit more detailed, especially in terms of whether model predictions have previously been compared to experimental PO2 values.

3. This study was performed on one type of rat skeletal muscle. Can the authors comment on whether the results should be applicable to other rat muscles and, potentially, to studies of human skeletal muscle? It seems it would mainly be a question of parameter values, rather than inherent physiological differences, but these might be more important than expected.

4. The authors present results (Fig.5) for the TA as a whole, and also separately for the oxidative core and glycolytic cortex. The two best capillary-based measures (LCD and D_max) show more relative importance (almost 10X) for the cortex than the core, which seems counter-intuitive. Is this just a mathematical artefact of the ML method used, or does it have some significance? Also, the TA as a whole seems to inherit the cortex results. Is this just a relative volume (or area) effect?

Minor Comments:

-Paragraphs 2 and 3 on P.14 have some grammatical errors (missing apostrophes).

-Although the meaning is fairly clear, the empty columns in Table 2 under Fibre Type could have the relevant parameter values from the first column repeated.

-Line 3 on P.19 states "the use a broader".

END OF COMMENTS

Response to the Referees

Referee #1:

This manuscript seeks to assess what capillary indices give the best reflection of tissue pO2. They used model calculations to assess the tissue pO2. The manuscript comes to the conclusion that the local capillary density and a new parameter, the maximal diffusion distance from a capillary in a capillary domain overlapping a fibre, are the best indicators of tissue oxygenation.

Fibre pO2 seems to be high in, for instance, figure 3, for a working muscle, where it has been suggested that the pO2 within a fibre maybe close to 1.5 mmHg during exercise, even when capillary pO2 is 30 mmHg.

Response: Firstly, we would like to thank you for your positive overview of our work.

We also thank the reviewer for this thoughtful observation regarding the physiological plausibility of the simulated fibre PO_2 values. We agree that in vivo measurements have shown that intracellular PO_2 in contracting muscle fibres can fall to very low values (≈ 1–2 mmHg) even when capillary PO_2 remains near 30 mmHg. The simulations presented in Figures 3-5 were performed under baseline metabolic demand, consistent with prior image-based modelling studies aimed at identifying geometric determinants of oxygen distribution under resting conditions.

To address this important point, we conducted a set of additional sensitivity analyses, repeating the entire image-based modelling and statistical workflow with tissue oxygen consumption ($\dot{M}O_2$) increased tenfold (×10) to represent an exercise-like, high-demand state. The corresponding results, presented in Figures A1–A3 in the Appendix, reproduce the analyses of Figures 3–5 under high metabolic demand. As expected, absolute fibre PO_2 values decreased substantially, consistent with experimental estimates for active muscle; however, the qualitative patterns, non-linear relationships, and predictor rankings among supply indices remained essentially unchanged. LCD and D_{max} continued to emerge as the strongest and most sensitive predictors of fibre oxygenation, hence confirming that our key findings are robust across a physiologically relevant range of oxygen demands.

We have now described this sensitivity analysis in the Methods, summarised the key results in the Results and Discussion sections, with cross-references to the new appendix figures and short notes added to the captions of Figures 3–5.

Also, suggestions have been made that fibre pO2 may not be best modelled with the Krogh cylinder, but rather the Hill model and that intracellular oxygen profiles are rather flat and metabolism not oxygen diffusion limited, as for instance realised by a 'mitochondrial powergrid' (e.g. review by David C Poole, Timothy I Musch in: Function, 4(3):zqad013, 2023: Capillary-Mitochondrial Oxygen Transport in Muscle: Paradigm Shifts). While I do not necessarily agree with the concepts proposed in that review, it would be good to discuss this and give some thoughts would affect your pO2 calculations and impacts of capillary indices on pO2.

The manuscript is well-written and a clear rationale for the work is given.

Response: We thank the reviewer for this insightful comment highlighting recent conceptual developments in models of oxygen transport and metabolic regulation. We agree that the work by Poole and Musch (Function, 4(3): zqad013, 2023) and others has re-evaluated the traditional Krogh-type paradigm, suggesting that intracellular PO_2 gradients may be minimal under physiological conditions due to a spatially integrated mitochondrial network ("mitochondrial powergrid") that minimizes diffusion limitation.

Our study, however, specifically addresses conditions in which oxygen diffusion becomes limiting—for example, in regions of reduced capillary density, impaired perfusion, or elevated metabolic demand—where the assumption of uniform intracellular PO_2 may not hold. Explicitly modelling diffusion allows us to quantify how capillary geometry and density influence tissue oxygenation under such regimes. Importantly, when tissue oxygen consumption ($\dot{M}O_2$) is increased tenfold, our simulations predict intracellular PO_2 levels (~1–2 mmHg; Appendix Figure 4) consistent with experimental values reported by Poole and colleagues, confirming the physiological validity of our approach.

We have now revised the "Potential Limitations" section to include a broader discussion of this conceptual debate, acknowledging both the "powergrid" framework and the continuing value of diffusion-based models for characterising structural determinants of tissue oxygenation. The new text (shown below) also clarifies the complementary relationship between diffusion models and future developments that may integrate mitochondrial topology and metabolic feedback.

"Recent work has challenged the traditional assumption of appreciable intracellular oxygen gradients and diffusion-limited metabolism, proposing instead that mitochondrial organization and intracellular oxygen transport may function as an integrated "powergrid" that maintains relatively uniform PO_2 across the fibre (Poole & Musch, 2023). While such frameworks may describe diffusion-sufficient conditions, our modelling explicitly targets scenarios where oxygen diffusion becomes limiting—for example, in tissues with reduced capillary density, impaired perfusion, or elevated metabolic demand. Under these circumstances, the assumption of spatially uniform intracellular PO_2 may not hold, and diffusion gradients become functionally significant. Incorporating explicit diffusion therefore enables quantification of how microvascular geometry and density influence both intracellular and interstitial oxygen availability. Consistent with experimental observations (Poole & Musch, 2023), our high-demand simulations predict intracellular PO_2 values within the physiological range observed during muscle contraction (Figure A4). Future developments could integrate finer-scale mitochondrial representations to explore how such "powergrid" mechanisms interact with oxygen diffusion and capillary geometry to sustain energetic homeostasis (Al-Shammari et al., 2019; Poole & Musch, 2023)."

Abstract

Line 5: There was not so much a comparison of capillary indices with fibre pO2, but rather which parameter(s) correlated best with the calculated fibre pO2.

Response: We agree that our analysis focused on identifying which indices most strongly correlate with (and predict) calculated fibre PO_2, rather than a direct comparison. We have re-worded the abstract to add clarity on the above point. The updated text now reads

" We assessed six local supply indices to determine which best correlated with and predicted calculated fibre PO_2 …"

Line 6 from bottom: Do you mean 'venous' capillary-level pO2?

Response: We thank the reviewer for raising this point. In the Abstract, "capillary-level PO_2" refers to the capillary source PO_2 specified at the tissue boundary (30 mmHg in our simulations) rather than to a venous or end-capillary value. As shown in Figure 3A, mean PO_2 in fibres with high local capillary supply (high LCD_i or low $D_{max,i}$) asymptotically approach this source PO_2. The resulting plateau arises because, under conditions of abundant capillary coverage, the oxygen gradient between the capillary and fibre becomes minimal and further increases in supply fail to elevate intracellular PO_2. Thus, the plateau represents a diffusion-equilibrium limit, not a venous saturation.

To clarify this, we revised the Abstract to read as follows:

"… to capture a curvilinear relationship that plateaus as mean fibre PO_2 approaches the capillary source PO_2 …",

and we added an explicit note in the Results & Discussion to reference a new supplementary figure (Figure A5 in the Appendix) showing a Hill-equation fit that quantitatively illustrate this nonlinear plateauing (or saturation).

Methods

Clearly described method. However, I do suggest changing the name 'fibre region' into the more commonly used 'fibre cross-sectional area'. I understand that 'area' is mentioned lower in the tables, but perhaps it is making matters unnecessarily complicated, and it could also be referred to as FCSA. Same for 'capillary domain' where I think you could as well write 'capillary domain area'.

Response: We thank the reviewer for this helpful suggestion and agree that the term fibre cross-sectional area (FCSA) is widely used in the histological literature. However, in the context of our image-based computational model, the term fibre region has a specific geometric meaning: it refers to the two-dimensional spatial set of tissue occupied by an individual fibre, not simply the scalar measure of its area.

This distinction is important for the mathematical definitions and formulas in Table 1, where we compute intersections/overlaps of 2D regions (for example, between fibre regions and capillary domains) before deriving their corresponding areas. Replacing region with area throughout would obscure this geometric meaning and could cause confusion in the interpretation of area-based supply measures.

To avoid ambiguity, we have clarified this distinction in the Methods and Table 1 captions, while continuing to use fibre region and capillary domain when referring to spatial (planar) entities, and fibre cross-sectional area (FCSA) or capillary domain area (CDA) when referring to their scalar measurements.

Can you discuss briefly how Mb in type IIa cells would affect the calculations? In table 2 it is suggested that there is no Mb in type IIa cells.

Response: We thank the reviewer for this important observation. To clarify, Table 2 specifies that Type IIa fibres were modelled with half the myoglobin (Mb) concentration of Type I fibres, and Type IIb/x fibres with one-tenth that value; none were modelled as lacking Mb.

Our simulations suggest that increasing Mb concentration in Type II fibres improves their mean fibre PO_2, but this effect scales directly with the level of oxygen demand ($\dot{M}O_2$) and inversely with capillary perfusion (capillary PO_2, P_{cap}). Myoglobin facilitation becomes significant primarily when intracellular PO_2 falls below its half-saturation pressure (~5 mmHg) - conditions that arise under very high metabolic demand or markedly reduced perfusion (low Pcap). Under normoxic or moderate conditions, the influence of Mb on fibre PO_2 is relatively marginal.

Importantly, varying Mb concentration across fibre types affects only the absolute values of PO_2 and does not materially alter the relationships between PO_2 and the local capillary-supply indices (LCD_i and $D_{max,i}$), which remain qualitatively consistent across fibre types and metabolic conditions. We have clarified this point in the Discussion, noting that differences in myoglobin concentration mainly influence absolute PO_2 under hypoxic conditions, while the geometric (spatial) determinants of oxygen supply remain dominant.

Referee #2:

This manuscript describes the results of a study that utilizes new histological data on rat skeletal muscle (tibialis anterior, TA) and computational modeling of oxygen (O2) transport (previously described OTM model) to evaluate various capillary-based measures of O2-transport efficiency. Both standard statistical and machine learning (ML) approaches are used to compare estimated muscle fiber oxygenation (O2 partial pressure, PO2) with predictions of the efficiency measures, which are based on 2D muscle sections where capillary, muscle fiber, and interstitial compartments are considered. Six measures are evaluated and two (the second of which I believe is novel) are found to strongly correlate with PO2, in particular, local capillary density (LCDi, positively correlated) and local capillary-fiber maximum distance (Dmaxi, negatively correlated). LCDi and D_maxi are shown to be informative both by standard statistics and several machine learning models, so the results are quite strong. I believe these results could have a significant impact in the field, and only have a few comments for the authors to consider, detailed below.

Response: Thank you for your positive evaluation of our work, below we have detailed responses to your comments, and made changes throughout the manuscript.

Major Comments:

According to Fig.3, the results presented focus on muscle fibers that have PO2 levels of 18mmHg or above. Given the assumed Pcrit of 0.5mmHg, this means that O2 consumption is barely impacted by decreased muscle PO2 (97% or greater of max). This presumably corresponds to healthy resting muscle, but it's not clear how generally applicable the results are to, for example, light or moderate exercise. Have the authors considered either increasing O2 consumption or decreasing the input capillary PO2 of 30mmHg in the computational model?

Response: We thank the reviewer for this insightful comment regarding the applicability of our findings beyond resting conditions. We confirm that our baseline simulations represent resting muscle.

To examine how our results extend to more metabolically demanding conditions, we conducted an additional sensitivity analysis in which tissue oxygen consumption ($\dot{M}O_2$) was increased tenfold (×10). This simulates a high-demand, exercise-like state. The results are now presented in Figures A1–A3 in the Appendix, and are described in the Methods, Results, and Discussion sections (see also our **Response to Reviewer 1, Comment #2**).

These simulations show that although absolute fibre PO_2 values decrease markedly—as expected under elevated metabolic demand—the qualitative relationships and relative rankings of capillary supply indices remain unchanged. In particular, LCD_i and $D_{max,i}$ continue to exhibit the strongest, nonlinear correlations with fibre PO_2, whereas integer-based indices fail to capture these features. This demonstrates that our principal findings are robust across a physiologically relevant range of oxygen supply and demand.

The description of the OTM model on P.9 (second paragraph) could be a bit more detailed, especially in terms of whether model predictions have previously been compared to experimental PO2 values.

Response: We thank the reviewer for this helpful suggestion. Regarding model validation, while OTM has not been directly compared with experimentally measured intracellular PO_2 on a fibre-by-fibre basis, its predictions under high metabolic demand ($\dot{M}O_2$ × 10-12; Appendix Figure 4) are quantitatively consistent with recent experimental observations of low intracellular PO_2 (~ 1-5 mmHg) and shallow intracellular gradients in contracting muscle (Poole & Musch, 2023). This correspondence demonstrates that the model yields PO_2 distributions consistent with experimentally observed physiological ranges, supporting its applicability to skeletal muscle oxygen transport.

We have expanded on this point in the Methods section to clarify the OTM's provenance, validation, and physiological grounding. Specifically, we now state in the Methods subsection "Image-based computational model of tissue PO_2" (first paragraph):

"Originally developed and described in detail by (Al-Shammari et al., 2019), the OTM was also validated against experimental microvascular morphometry and later shown to reproduce physiologically realistic tissue and fibre PO_2 distributions. In particular, the model's predictions under high oxygen demand (~1–5 mmHg; Figure A4) are consistent with experimental estimates of intracellular PO_2 during exercise (Poole & Musch, 2023), supporting its physiological validity."

This study was performed on one type of rat skeletal muscle. Can the authors comment on whether the results should be applicable to other rat muscles and, potentially, to studies of human skeletal muscle? It seems it would mainly be a question of parameter values, rather than inherent physiological differences, but these might be more important than expected.

Response: We thank the reviewer for this valuable point. The tibialis anterior (TA) muscle was selected precisely because it contains two physiologically distinct compartments—an oxidative core and a glycolytic cortex—which differ substantially in fibre type composition, capillary density, and cross-sectional geometry. This internal heterogeneity provides an ideal system for examining how morphometric indices behave across a wide range of phenotypes within the same muscle.

The TA cortex, composed primarily of a single fast-twitch fibre type with relatively uniform size, is structurally similar to homogeneous muscles such as the soleus or myocardium. In contrast, the oxidative core represents a mixed-fibre region with large variability in both fibre cross-sectional area and capillary domain area. By analysing these contrasting regions, our study effectively spans the physiological extremes of muscle architecture found in locomotory muscles.

Although parameter values (e.g., capillary PO_2, diffusivity, metabolic rate) may vary among muscles and species, the qualitative relationships we observe—particularly the nonlinear "dependence" of fibre PO_2 on area-based supply indices (LCD_i and $D_{max,i}$)—are grounded in geometric and diffusive principles. Similar heterogeneity and capillary-fibre organization occur in both rodent and human skeletal muscle (Egginton, 1990; Zeller-Plumhoff et al., 2017), so we expect comparable behaviour.

To emphasise this generality and to clarify applicability, we have:

1. Updated Figure 3 to display data points from the TA core and cortex in different colours, illustrating that both regions follow the same curvilinear trends between local capillary supply indices and calculated fibre PO_2.
2. Inserted a new paragraph at the end of the "**Correlation with fibre PO_2**" subsection (Results) describing the core–cortex comparison.
3. Inserted a sentence in the Discussion (after the paragraph beginning "Our findings demonstrate that area-based indices…") to note that these relationships were consistent across muscle compartments and likely extend to other mammalian muscles.

4. The authors present results (Fig.5) for the TA as a whole, and also separately for the oxidative core and glycolytic cortex. The two best capillary-based measures (LCD and D_max) show more relative importance (almost 10X) for the cortex than the core, which seems counter-intuitive. Is this just a mathematical artefact of the ML method used, or does it have some significance? Also, the TA as a whole seems to inherit the cortex results. Is this just a relative volume (or area) effect?

Response: We thank the reviewer for this perceptive question, which indeed relates to the underlying heterogeneity between the oxidative core and glycolytic cortex discussed above. The key point is that integer-based indices remain insensitive to this structural heterogeneity, whereas area-based indices (LCD_i and $D_{max,i}$) consistently capture the most physiologically meaningful relationships.

Regarding the apparent difference in magnitude of relative importance between compartments (Figure 5), this is a consequence of the machine-learning (ML) normalisation, rather than a biological effect. Each model (core, cortex, whole TA) was trained independently and standardised within its own dataset. Consequently, the absolute scales of variable-importance scores are not directly comparable across panels, and interpretations/comparisons should only be made within each figure.

The similarity between the whole-muscle and cortical results is primarily a relative volume effect, since the cortex constitutes a larger proportion of the TA cross-section and therefore exerts a greater influence on the combined dataset. We have clarified both points in the Figure 5 caption and added a note in the Results to alert readers to this ML normalisation-related effect.

Minor Comments:

-Paragraphs 2 and 3 on P.14 have some grammatical errors (missing apostrophes).

Response: We have carefully reviewed these paragraphs for grammatical errors and corrected the missing apostrophes in the revised manuscript.

-Although the meaning is fairly clear, the empty columns in Table 2 under Fibre Type could have the relevant parameter values from the first column repeated.

Response: We have amended our table to include the individual values for each fibre type.

-Line 3 on P.19 states "the use a broader".

Response: We have fixed this typo in the revised manuscript. It now reads “the use of a broader”.

Dear Dr Al-Shammari,

Re: JP-RP-2025-288043R1 "Utility of local capillary supply indices: insights from computational image-based 1 modelling" by Abdullah A Al-Shammari, Roger W.P Kissane, Ali Ashkanani, Ashraf Madhoun, Fahd Al-Mulla, Eamonn A Gaffney, and Stuart Egginton

We are pleased to tell you that your paper has been accepted for publication in The Journal of Physiology.

The last Word (or similar) version of the manuscript provided will be used by the Production Editor to prepare your proof. When this is ready you will receive an email containing a link to Wiley's Online Proofing System. The proof should be thoroughly checked and corrected as promptly as possible.

Authors should note that it is too late at this point to offer corrections prior to proofreading. Major corrections at proof stage, such as changes to figures, will be referred to the Editors for approval before they can be incorporated. Only minor changes, such as to style and consistency, should be made at proof stage. Changes that need to be made after proof stage will usually require a formal correction notice.

All queries at proof stage should be sent to: TJP@wiley.com.

Following copyediting and typesetting the accepted version will be published online.

Yours sincerely,

Natalia Trayanova
Senior Editor
The Journal of Physiology

IMPORTANT POINTS TO NOTE FOLLOWING ACCEPTANCE OF YOUR PAPER:

• IMPORTANT NOTICE ABOUT OPEN ACCESS: To assist authors whose funding agencies mandate immediate public access to published research findings, The Journal of Physiology allows authors to pay an Open Access (OA) fee to have their papers made freely available immediately on publication.

The Corresponding Author will receive an email from Wiley with details on how to register or log-in to Wiley Authors Services where you will be able to place an order.

• You can check if your funder or institution has a Wiley Open Access Account here: https://authorservices.wiley.com/author-resources/Journal-Authors/licensing-and-open-access/open-access/author-compliance-tool.html.

• TRANSPARENT PEER REVIEW POLICY: To improve the transparency of its peer review process, The Journal of Physiology publishes online as supporting information the peer review history of all articles accepted for publication. Readers will have access to decision letters, including Editors' comments and referee reports, for each version of the manuscript as well as any author responses to peer review comments. Referees can decide whether or not they wish to be named on the peer review history document.

• You can help your research get the attention it deserves! Check out Wiley's free Promotion Guide for best-practice recommendations for promoting your work at: www.wileyauthors.com/eeo/guide. You can learn more about Wiley Editing Services which offers professional video, design, and writing services to create shareable video abstracts, infographics, conference posters, lay summaries, and research news stories for your research at: www.wileyauthors.com/eeo/promotion.

• If you would like to receive our 'Research Roundup', a monthly newsletter highlighting the cutting-edge research published in The Physiological Society's family of journals (The Journal of Physiology, Experimental Physiology, Physiological Reports, The Journal of Nutritional Physiology and The Journal of Precision Medicine: Health and Disease), please click this link, fill in your name and email address and select 'Research Roundup':
https://www.physoc.org/journals-and-media/membernews

EDITOR COMMENTS

The revision has addressed the reviewer's concerns and now has potential to make an impact in the field.

REFEREE COMMENTS

Referee #2:

I feel the revised manuscript is substantially improved in terms of clarity and the strength of its conclusions, particularly its relevance to both resting and working muscle, and its potential applicability to a wide range of skeletal muscles. I have no further comments for the authors to address.

****
